# I226R Protein of African Swine Fever Virus Is a Suppressor of Innate Antiviral Responses

**DOI:** 10.3390/v14030575

**Published:** 2022-03-11

**Authors:** Jinxuan Hong, Xiaojuan Chi, Xu Yuan, Faxin Wen, Kul Raj Rai, Lei Wu, Zhongbao Song, Song Wang, Guijie Guo, Ji-Long Chen

**Affiliations:** Key Laboratory of Fujian-Taiwan Animal Pathogen Biology, College of Animal Sciences, Fujian Agriculture and Forestry University, Fuzhou 350002, China; jinxuanhong95@163.com (J.H.); chixiaojuan88@126.com (X.C.); ylyf1025@163.com (X.Y.); wenx1008@163.com (F.W.); kulrajrai701@gmail.com (K.R.R.); 18846429829@163.com (L.W.); virus_szb@163.com (Z.S.); wscookie@163.com (S.W.); guojie1125@163.com (G.G.)

**Keywords:** ASFV, I226R, innate immunity, NF-κB, IRF3

## Abstract

African swine fever is one of the most devastating swine diseases caused by African swine fever virus (ASFV). Although ASFV encodes more than 160 viral proteins, the implication of a majority of ASFV proteins in regulating host immunity is yet to be explored, and the mechanisms of immune evasion by ASFV proteins are largely unknown. Here, we report that the I226R protein of ASFV significantly suppressed innate immune responses. The ectopic expression of ASFV I226R in 293T cells significantly inhibited the activation of interferon-stimulated response element promoters triggered by Sendai virus (SeV), poly(I:C), or cyclic GMP-AMP synthase (cGAS)/STING. The I226R protein caused a significant decrease in the expression of interferons and interferon-stimulating genes in cells infected with SeV. Similar results were obtained from experiments using I226R-overexpressed PK15 and 3D4/21 cells stimulated with vesicular stomatitis virus. We observed that I226R inhibited the activation of both nuclear factor-kappa B (NF-κB) and interferon regulatory factor 3 (IRF3). Furthermore, it was shown that overexpression of I226R suppressed IRF3 activation and caused the degradation of NF-κB essential modulator (NEMO) protein. The I226R-induced NEMO degradation could be prevented by treatment with MG132, a proteasome inhibitor. Together, these results reveal that the ASFV I226R protein impairs antiviral responses, likely through multiple mechanisms including the suppression of NF-κB and IRF3 activation, to counteract innate immune responses during the viral infection.

## 1. Introduction

African swine fever virus (ASFV) is the only member of Asfaviridae family, which can cause a highly lethal infectious disease in domestic pigs and wild boars [1,2], leading to huge economic losses in the swine industry worldwide and threatening food security. African swine fever (ASF) caused by ASFV is classified as a notifiable disease by the World Organisation for Animal Health. ASF was first described in East Africa (Kenya Colony) in 1921 [3]. Since then, the disease has become widespread in Africa, Europe, and Asia [4]. ASF was first reported in China in August 2018, and quickly swept across China in the next few months [5]. Outbreaks of ASF have also been reported in other Asian countries, such as Vietnam, North Korea, and Laos, since 2018 [6,7,8,9]. Extensive research to develop ASF vaccines and antiviral drugs has been conducted; however, no commercial products are available yet, posing a great challenge for global ASF prevention [10,11]. 

ASFV is a complex double-stranded linear DNA virus with icosahedral symmetry [12]. The genomic length of ASFV ranges from 170 to 193 kb, and the genome can encode a large number of viral proteins (~160 proteins) [13,14]. Previous studies have demonstrated that ASFV infection causes severe immunosuppression in its natural hosts, resulting in successful infection and viral replication [15]. Several ASFV proteins have been shown to regulate innate immunity. For instance, the A238L protein of ASFV can inactivate nuclear factor-kappa B (NF-κB) [16,17,18]; MGF505-7R dampens the production of interferon-β (IFN-β) [19]; and DP96R exerts an immunosuppressive effect by reducing the phosphorylation of TANK-binding kinase 1 (TBK1), thereby inhibiting the activation of downstream interferon regulatory factor 3 (IRF3) [20]. Therefore, the immunosuppressive mechanism could be attributed to the subversion of innate immunity by viral proteins. Although several viral proteins are revealed to play a key role in the circumvention of host innate immunity, the implication of a majority of ASFV proteins in regulating innate immune responses is yet to be characterized. Moreover, the molecular mechanisms by which these proteins are involved in the regulation of innate immunity are poorly understood.

Innate immunity is a critical first line of defense against viral infection [21,22]. The host is armed with pattern-recognition receptors (PRRs) to sense pathogen-associated molecular patterns (PAMPs). Toll-like receptors (TLRs), C-type lectin receptors (CLRs), NOD-like receptors (NLRs), RIG-I-like receptors (RLRs), and DNA-associated receptors are important PRRs to detect PAMPs such as viral DNA, RNA, and replication intermediates [23,24,25]. Sensing PAMPs by PRRs activates innate immune signaling pathways. Cyclic GMP-AMP synthase (cGAS) is a well-characterized PRR responsible for sensing DNA viruses [26]. DNA sensing by cGAS leads to the generation of cyclic GMP-AMP (cGAMP) in the presence of ATP and GTP. Then, cGAMP binds to the stimulator of interferon gene-encoded protein (STING) to recruit TBK1 to form a complex that activates the transcription factors, including NF-κB and IRF3 [27]. Activated transcription factors translocate into the nucleus to induce robust expression of various cytokines and chemokines.

NF-κB is a crucial component of innate immunity in mediating cytokine and chemokine production. In mammals, there are five NF-κB family members, including p65 (RelA), RelB, c-Rel, p50/p105 (NF-κB1), and p52/p100 (NF-κB2) [28]. All members of the NF-κB family share a conserved domain called the Rel homology domain (RHD). A virus infection activates the inhibitor of kappa B kinase (IKK), which contains catalytic subunits IKKα, IKKβ, and regulatory subunits IKKγ (NEMO), leading to IκBα ubiquitination and the proteasomal degradation of IκBα, followed by NF-κB release. The released NF-κB translocates into the nucleus and drives the transcription of NF-κB target genes [29]. Similar to NF-κB, interferon regulatory factors (IRFs) also play an important role in interferon production. IRF3 is activated by phosphorylation through kinases TBK1 and IKKε, leading to the dimerization and translocation of IRF3/7 homo- or hetero-dimers to the nucleus, and finally, transcriptional activation of the IFN promoter [30]. Since viral proteins are implicated in the modulation of innate immunity for efficient virus replication, identifying the viral proteins that counter critical components of the innate immune system provides an important theoretical basis for the development of effective vaccines and antiviral drugs.

This study aimed to identify and characterize the ASFV proteins involved in regulating host innate immunity. We screened ten proteins and found that the I226R protein of ASFV suppressed antiviral innate immunity. The ASFV I226R protein is about 27kd encoded by the viral *I226R* gene. The presence of the *I226R* gene was first reported in 1996 by transcriptional analysis [31]. Thereafter, a subsequent report revealed that the I226R protein could be expressed in the early and late stages of viral infection [32]. Recently, it was reported that I226R knockout attenuated ASFV in pigs. Moreover, pigs infected with I226R-deleted virus were resistant to infection by its virulent parental virus, indicating that I226R is a promising candidate to design recombinant vaccines for genotype II ASF [33]. However, the biological function of the I226R protein is largely unknown. In this study, we reveal that the ASFV I226R protein can antagonize innate immunity by inhibiting the activation of NF-κB and the IRF3 signaling pathway. Our findings uncover a new mechanism of how ASFV evades innate immunity, and provide a new theoretical basis in designing a recombinant vaccine.

## 2. Materials and Methods

### 2.1. Cells, Virus and Plasmids

For this study, 293T, MDCK, PK15, 3D4/21 cells were purchased from American Type Culture Collection (ATCC). The 293T, MDCK, and PK15 cells were maintained in Dulbecco’s Modified Eagle’s Medium (DMEM, Gibco, Thermo Fisher Scientific, Inc., Waltham, MA, USA), supplemented with 10% fetal bovine serum (Gibco, Thermo Fisher Scientific, Inc., Waltham, MA, USA), penicillin (100 U/mL), and streptomycin (100 μg/mL). The 3D4/21 cells were maintained in Roswell Park Memorial Institute 1640 Medium (Gibco, Thermo Fisher Scientific, Inc., Waltham, MA, USA), containing 10% FBS, 1% nonessential amino acids (Invitrogen, Carlsbad, CA, USA), 100 U/mL penicillin, and 100 μg/mL streptomycin. All cells were cultured at 37 °C under 5% CO_2_ atmosphere, as previously described [34].

Sendai virus (SeV) was generated and propagated in specific-pathogen-free chicken embryos, as previously described [35]. The virus titer was determined by a hemagglutination test, as described previously [36]. Vesicular stomatitis virus (VSV) was preserved in our laboratory and propagated in PK15 cells.

The A137R, K145R, A151R, KP177R, K205R, I215L, I226R, I243L, E248R, or C257L coding sequence of the ASFV isolate China/2018/AnhuiXCGQ (Gen-108 Bank accession number: MK128995.1), fused with the FLAG tag, was optimized and synthesized by Genewiz Biotechnology Co., Ltd. (Suzhou, China) and cloned into a pcDNA3.1(−) vector. Recombinant plasmids were confirmed by restriction digestion and sequencing. Other human genes (RIG-I, MAVS, RIP1, TRAF2, TRAF6, TAK1, IRF3), fused with specific peptide tags, were also cloned into indicated vectors, as described previously [37]. The plasmids IKKβ-Flag, p65-Flag, and NEMO-Flag were purchased from Wuhan Miaoling Biotechnology Co., Ltd. (Wuhan, China).

The luciferase reporter plasmids used in this study included an IFN-β promoter reporter plasmid (IFN-β-Luc), carrying promoter sequences of IFN-β used to examine the activation of the IFN-β promoter; an NF-κB-Luc plasmid, harboring NF-κB-binding elements used to detect the activation of NF-κB; a (pRDIII-I)4-Luc plasmid, carrying IRF3-binding elements used to examine the activity of IRF3; and a control reporter plasmid, pRL-TK. All these reporter plasmids were gifts from Dr. Chunfu Zheng (Fujian Medical University, Fuzhou, China) [38,39]. An ISRE luciferase reporter plasmid (ISRE-Luc), harboring interferon (IFN)-stimulated response elements used to detect IFN production and secretion, was purchased from YEASEN Biotechnology Co., Ltd. (Shanghai, China).

### 2.2. Antibodies and Reagents

The lysosome inhibitors chloroquine (CQ), NH_4_Cl, and mouse anti-Flag monoclonal antibody were purchased from Sigma-Aldrich (St. Louis, MO, USA). The protein synthesis inhibitor cycloheximide (CHX), the proteasome inhibitor MG132, and rabbit anti-NF-κB p65 and anti-phospho-NF-κB p65 (Ser536) polyclonal antibodies were purchased from Cell Signaling Technology (Boston, MA, USA). Mouse anti-β-actin and anti-phospho-IRF3 monoclonal antibodies were obtained from Abcam (Cambridge, UK). Mouse anti-HA polyclonal antibody and anti-Myc monoclonal antibody and rabbit anti-IRF3 and anti-Flag polyclonal antibodies were purchased from Proteintech (Wuhan, China).

### 2.3. Dual-Luciferase Reporter Assay

The 293T cells were sub-cultured overnight to 80% confluence. The IFN-β-Luc, NF-κB-Luc, or (pRDIII-I)4-Luc plasmid was co-transfected with the pRL-TK and indicated plasmids using VigoFect (Vigorous Biotechnology, Beijing, China) according to the manufacturer’s instruction. At 24 h post-transfection, the cells were challenged with 100 HAU/mL SeV for 16 h, followed by dual-luciferase activity assays using a dual-luciferase reporter assay kit according to the manufacturer’s instruction (Promega Corp., Madison, WI, USA) [38,39]. The 293T cells were co-transfected with the NF-κB-Luc reporter plasmid, the pRL-TK and I226R-Flag plasmid, or EV, along with RIG-I, MAVS, RIP1, TRAF2, TRAF6, IKKβ, or p65 plasmids. The cells were collected after 24 h, followed by dual-luciferase activity assays using a dual-luciferase reporter assay kit.

### 2.4. Enzyme-Linked Immunosorbent Assay (ELISA)

To quantify the secretion of IFN-β in response to SeV infection, the cell supernatant was collected and assayed with ELISA using a human IFN-β ELISA kit (eBioscience, San Diego, CA, USA) according to the manufacturer’s instruction [35].

### 2.5. RNA Preparation, RT-PCR and RT-qPCR 

Total RNA was extracted from transfected or infected cells using Total RNA Kit I (Omega Bio-tek, Shenzhen, China) and used for cDNA synthesis by a HiScript III 1st Strand cDNA Synthesis Kit (Vazyme, Nanjing, China). Then, the cDNA was used for PCR or qPCR by Taq DNA polymerase (TaKaRa Bio Inc., Otsu, Japan) or qPCR SuperMix (TransGen Biotech, Beijing, China). The sequences of the primers used are available upon request. GAPDH was used as a reference housekeeping gene for internal standardization. For quantification, the 2-ΔΔCt methods were used to calculate the relative RNA levels against the GAPDH [34].

### 2.6. Plaque Assay

At 24 h post-transfection, the 293T cells were infected with influenza virus (PR8∆NS1) for 24 h, and the culture supernatant was collected for plaque assay, as previously described [40,41]. Briefly, the MDCK cells were infected with serial dilutions of the supernatants for 1 h; then, the cells were washed three times with PBS and overlaid with α-minimal essential medium containing 1.5% low-melting-point agarose and 2 μg/mL TPCK (l-1-tosylamido-2-phenylethyl chloromethyl ketone)-treated trypsin. Plaques were counted after 72 h incubation at 37 °C.

### 2.7. Generation of Stable Cell Lines

Cell lines overexpressing the I226R-Flag or an empty vector were generated, as previously described [37]. Briefly, lentiviruses encoding the I226R-Flag or an empty vector were produced in the 293T cells by co-transfecting with a pNL-I226R-Flag, a pNL-VSVG, and a pNL-package. The supernatant containing the lentiviruses was collected at 48 h post-transfection; then, it was used to infect PK15 or 3D4/21 cells to generate cell lines stably expressing I226R.

### 2.8. Immunofluorescence Assay

To determine the intracellular distribution of p65, immunofluorescence staining was performed, as described [42]. Briefly, virus-infected and plasmid-transfected cells were fixed with 4% paraformaldehyde for 15 min at room temperature (RT); then, they were permeabilized with 0.1% Triton-100 for exactly 10 min. After washing, the cells were blocked with 1% BSA-PBS for 1 h at RT; then, they were incubated with anti-p65 antibody for 1 h at 37 °C, followed by incubation with Alex Fluor 594-labeled secondary antibody for 1 h at 37 °C. The cells were rinsed again, and the nuclei were stained with DAPI for 10 min at RT. The cells were observed using a confocal laser-scanning microscope (LSCMFV500, Olympus, Japan) and analyzed with NIS Elements F 2.30 466 software.

### 2.9. Immunoprecipitation Assay

Immunoprecipitation was performed, as previously described [35]. Briefly, the transfected cells were washed three times with PBS and incubated with a cell lysis buffer (Beyotime Biotechnology, Haimen, China) for 10 min on ice. The supernatants were collected and transferred to fresh tubes on ice; then, 1 g of rabbit IgG and 20 L of protein A/G agarose (Beyotime Biotechnology, Haimen, China) were added to each tube and incubated for 1 h at 4 °C. The collected supernatants and a rabbit anti-Flag (1:1000) antibody were added to each tube for incubation for 1 h at 4 °C. Then, 40 µL protein A/G agarose was added to each tube for incubation at 4 °C overnight. The immunoprecipitated proteins were collected by centrifugation at 1000× *g* for 5 min at 4 °C after washing three times, and used for Western blotting assay.

### 2.10. Statistical Analysis

Statistical significance was determined by the Student’s *t*-test. Three independent experiments were performed with at least two technical replicates. All data represent the mean ± SD, and *p* values < 0.05 were considered to be statistically significant.

## 3. Results

### 3.1. I226R Protein Is a Key Inhibitor of IFN-β Response

**Figure 1:**
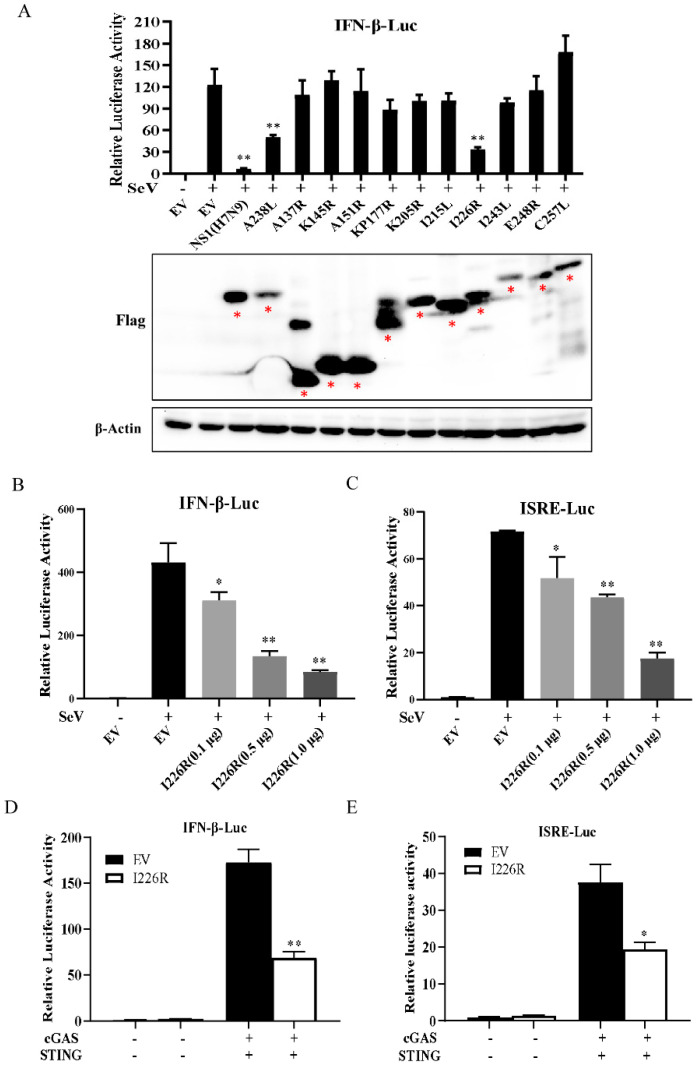
The I226R protein is a key inhibitor of IFN-β response: (**A**) 293T cells were co-transfected with 500 ng IFN-β-Luc, 50 ng pRL-TK, and 500 ng empty vector (EV) or other expression plasmids encoding the indicated ASFV viral proteins. After 24 h of co-transfection, cells were infected with SeV. Then, dual-luciferase activity was examined at 16 hpi. Western blotting was performed to examine the expression of candidate ASFV proteins. (**B**,**C**) 293T cells were co-transfected with 500 ng IFN-β-Luc (**B**) or ISRE-Luc (**C**), 50 ng pRL-TK, and I226R-Flag plasmid (0.1, 0.5, or 1.0 μg) or EV for 24 h, and infected with SeV for 16 h. Dual-luciferase activity assay was performed. (**D**,**E**) 293T cells were co-transfected with 300 ng IFN-β-Luc (**D**) or ISRE-Luc (**E**), along with 30 ng pRL-TK, 300 ng cGAS-Flag and 50 ng STING-HA, and 300 ng I226R-Flag or EV and for 24 h; then, dual-luciferase activity assay was performed. All the results are expressed as the means ± standard deviation from three independent experiments. Statistical analysis was performed using the Student’s *t*-test. * *p* < 0.05; ** *p* < 0.01.

In order to screen the ASFV proteins that could potentially inhibit the expression of IFN, we utilized dual-luciferase reporter assays. First, we synthesized ten ASFV genes that encode proteins expressed early in or at the mid-term of the viral life cycle, and cloned these genes into the vectors indicated: namely, A137R, K145R, A151R, KP177R, K205R, I215L, I226R, I243L, E248R, and C257L. We found that all these viral proteins could be expressed in 293T cells (Figure 1A). To test which protein could regulate the IFN-β promoter activity, 293T cells were transfected with the IFN-β-Luc and pRL-TK, with a plasmid carrying one of these ASFV genes or an empty vector. Because it is known that the influenza A virus NS1 and ASFV A238L proteins are potent inhibitors of IFN signaling [17,18,43], we used NS1 and A238L proteins as a positive control and the empty vector (EV) as the negative control. As shown in Figure 1A, among the ten ASFV proteins tested, the I226R protein most significantly inhibited the activation of the IFN-β promoter. In addition, we compared the inhibitory effect of I226R with that of A238L on the IFN-β luciferase using data normalization based on protein expression levels. Our results indicated that I226R and A238L had similar abilities to repress the IFN response (Supplementary Figure S1A). To further confirm the inhibitory effect of the I226R protein on IFN signaling, we evaluated the effect of the I226R protein on the IFN response induced by SeV infection or cGAS/STING expression using dual-luciferase reporter assays. We observed that the I226R protein significantly inhibited the activation of the IFN-β promoter and the ISRE reporter in a dose-dependent manner following infection with SeV. Moreover, the I226R protein also inhibited the activation of the IFN-β promoter and the ISRE reporter induced by cGAS/STING expression (Figure 1B–E). These results suggest that I226R is a potential candidate implicated in the suppression of IFN signaling during viral infection.

### 3.2. I226R Protein Reduces the Expression of IFNs Induced by SeV and Poly(I:C) 

**Figure 2:**
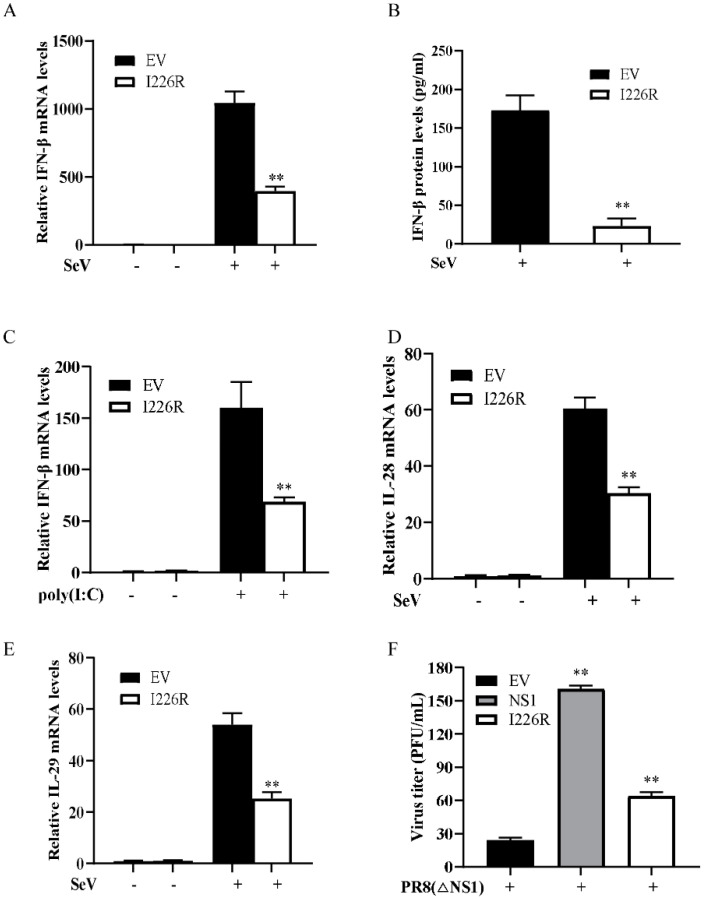
The I226R protein reduces the expression of IFNs induced by SeV and poly(I:C): (**A**–**C**) 293T cells were transfected with 3 μg I226R-Flag plasmid or EV for 24 h; then, they were infected with SeV (**A**,**B**), or were transfected with 1 μg/mL poly(I:C) (**C**) for 12 h. RT-qPCR and ELISA were performed to examine the expression of IFN-β mRNA and protein levels, respectively. (**D**,**E**) 293T cells were transfected with 3 μg I226R-Flag plasmid or EV for 24 h; then, they were infected with SeV for 12 h. Expression levels of IL28 (**D**) and IL29 (**E**) were measured by RT-qPCR analysis. (**F**) 293T cells were transfected with 3 μg EV, NS1(H7N9), or I226R-Flag plasmid for 24 h; then, they were infected with PR8 ∆NS1 (MOI = 1) for 12 h. The supernatant of virus-infected cells was used for plaque assay. All the results are expressed as the means ± standard deviation from three independent experiments. Statistical analysis was performed using the Student’s *t*-test. ** *p* < 0.01.

To further evaluate the antagonistic role of the I226R protein in innate immunity, we analyzed the effect of the I226R protein on the expression of IFN-β in 293T cells stimulated with SeV or poly(I:C). The 293T cells transfected with an EV or the I226R-Flag plasmid were infected with SeV. The expression of IFN-β was examined using ELISA and RT-qPCR, respectively. Consistent with the results from the dual-luciferase reporter assays, overexpression of the I226R protein caused a significant decrease in the virus-induced expression of IFN-β compared with control cells after SeV infection (Figure 2A,B). Next, we stimulated the 293T cells through transfection with poly(I:C), a synthetic analog of double-stranded RNA. Similarly, the I226R protein overexpression significantly inhibited the IFN-β production induced by poly(I:C) (Figure 2C). Moreover, we tested the expression of type III IFNs, including IL-28 and IL-29. Overexpression of I226R inhibited the virus-induced production of IL-28 and IL-29 (Figure 2D,E). Since the influenza A virus (IAV) NS1 protein is a potent inhibitor of type I IFN signaling, we studied the effect of the I226R protein on NS1-deleted IAV (PR8∆NS1) replication. For this, the 293T cells were transfected with I226R, IAV NS1, or an EV and infected with PR8∆NS1 virus. The virus titer was examined. Interestingly, the virus titer significantly increased after overexpression of the I226R or NS1 protein (Figure 2F). These data suggest that the I226R protein of ASFV can exert a potent inhibitory effect on IFN expression similar, to some extent, to the NS1 protein of the influenza virus.

### 3.3. I226R-Overexpressing Cells Have Impaired Expression of Several Critical ISGs

**Figure 3:**
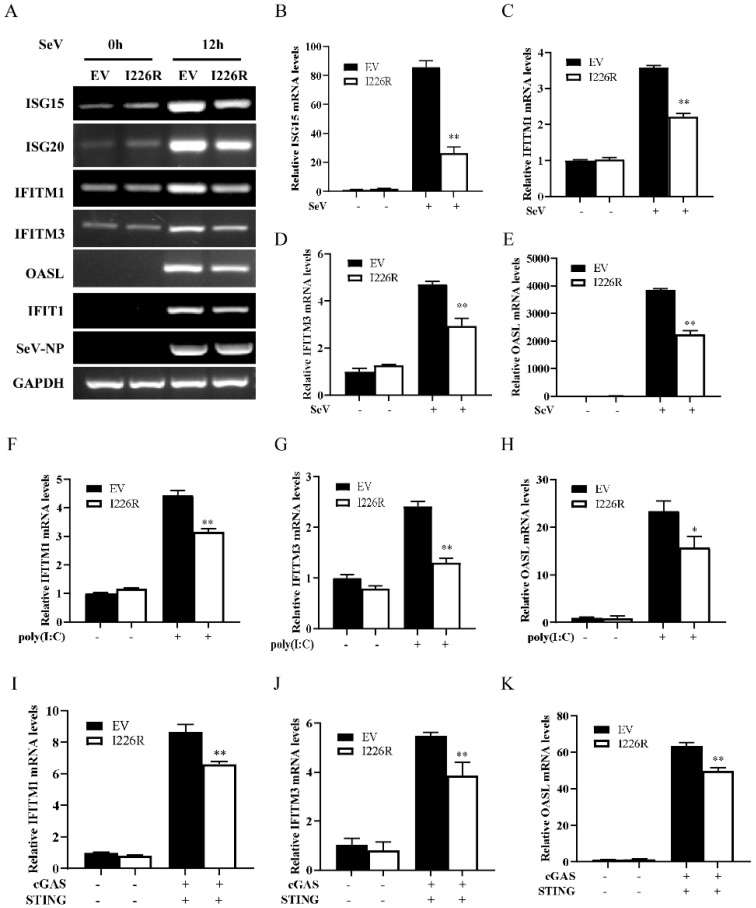
I226R-overexpressing cells have impaired expression of several critical ISGs. (**A**–**E**) 293T cells were transfected with 3 μg I226R-Flag plasmid or EV for 24 h; then, they were infected with SeV for 12 h. RT-PCR (**A**) and RT-qPCR analysis (**B**–**E**) were performed to examine the mRNA levels of ISG15 (**B**), IFITM1 (**C**), IFITM3 (**D**), and OASL (**E**). (**F**–**H**) 293T cells were transfected with 3 μg I226R-Flag plasmid or EV for 24 h; then, they were transfected with 1 μg/mL of poly(I:C) for 12 h. RT-qPCR analysis was performed to examine the mRNA levels of IFITM1 (**F**), IFITM3 (**G**), and OASL (**H**). (**I**–**K**) 293T cells were co-transfected with 2 μg I226R-Flag plasmid or EV and 500 ng cGAS-Flag and 100 ng STING-HA plasmids. RT-qPCR analysis was then performed to examine the mRNA levels of IFITM1 (**I**), IFITM3 (**J**), and OASL (**K**) 24 h later. The results are expressed as the means ± standard deviation from three independent experiments. Statistical analysis was performed using the Student’s *t*-test. * *p* < 0.05; ** *p* < 0.01.

Since ISGs are the important downstream antiviral effectors orchestrated by IFN pathways, we next examined the effect of the I226R protein on ISG expression induced by SeV infection and poly(I:C) treatment. We first examined the expression of ISGs, such as ISG15, ISG20, IFITM1, IFITM3, OASL, and IFIT1, in the cells overexpressing I226R or in EV control cells following SeV infection. As expected, the expression of the I226R protein led to the diminished expression of ISGs induced by SeV infection (Figure 3A–E and Figure S1B,C). Similarly, overexpression of the I226R protein also reduced the expression of some ISGs, such as IFITM1, IFITM3, OASL, IFIT1, and IFIT2, in cells stimulated through transfection with poly(I:C) (Figure 3F–H and Figure S1D,E). In addition, we observed that the I226R protein caused a decrease in the expression of IFITM1, IFITM3, OASL, and ISG15 upon stimulation by cGAS/STING (Figure 3I–K and Figure S1F). These results suggest that the I226R protein suppresses host innate immunity, likely through negatively regulating the expression of IFNs and thereby reducing the levels of some critical ISGs.

### 3.4. I226R Protein Inhibits VSV-Induced Expression of IFN-β and ISGs in Swine Cells

**Figure 4:**
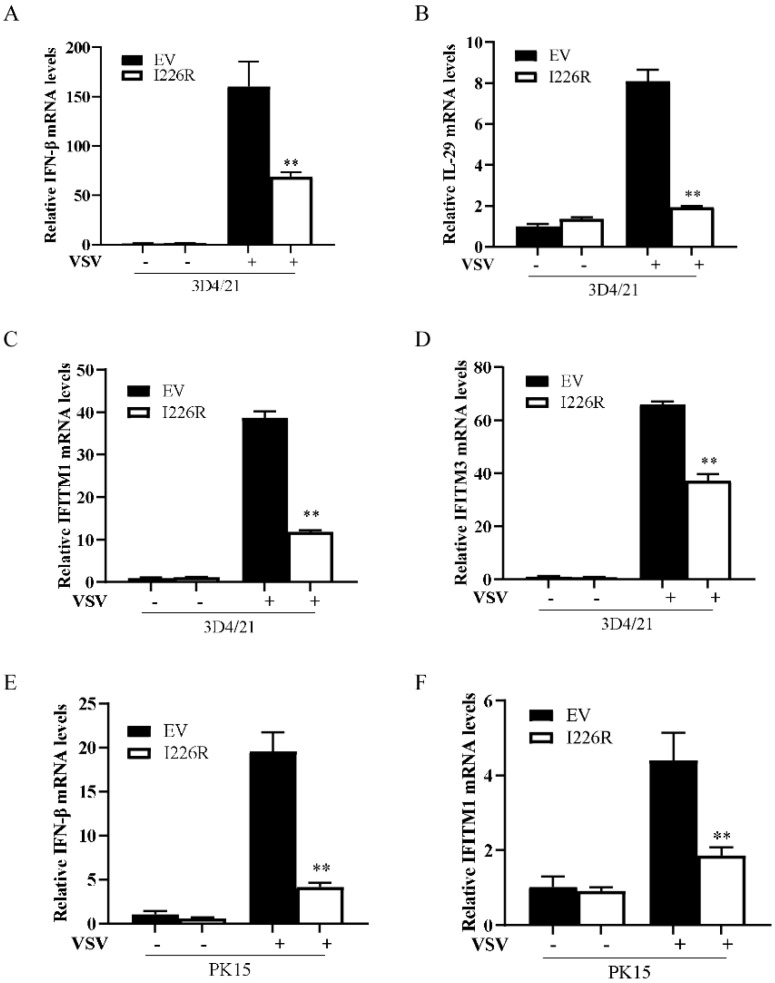
The I226R protein inhibits VSV-induced expression of IFN-β and ISGs in swine cells: (**A**–**D**) 3D4/21 cells stably expressing I226R or EV were generated; then, they were infected with VSV (MOI = 1) for 12 h. RT-qPCR analysis was performed to examine the mRNA levels of IFN-β (**A**), IL29 (**B**), IFITM1 (**C**), and IFITM3 (**D**). (**E**,**F**) PK15 cells stably expressing I226R or EV were generated; then, they were infected with VSV (MOI = 1) for 12 h. RT-qPCR analysis was performed to detect the mRNA levels of IFN-β (**E**) and IFITM1 (**F**). All the results are expressed as the means ± standard deviation from three independent experiments. Statistical analysis was performed using the Student’s *t*-test. ** *p* < 0.01.

Next, we further investigated the function of the I226R protein in suppressing host antiviral responses in swine cells. PK15 and 3D4/21 cells stably expressing I226R or an EV were infected with VSV, and the levels of IFN-β and ISGs were examined. The forced expression of the I226R protein in 3D4/21 cells caused a significant decrease in the production of VSV-induced IFN-β, IL-29, IFITM1, and IFITM3 compared with control cells after VSV infection (Figure 4A–D). Similarly, the expression of the I226R protein diminished the expression of IFN-β and IFITM1 induced by VSV infection in PK15 cells (Figure 4E,F). These data indicate that the I226R protein of ASFV inhibits the expression of IFN-β and ISGs in swine cells after VSV infection.

### 3.5. I226R Protein Impairs the Activation of NF-κB and IRF3 Signaling

**Figure 5:**
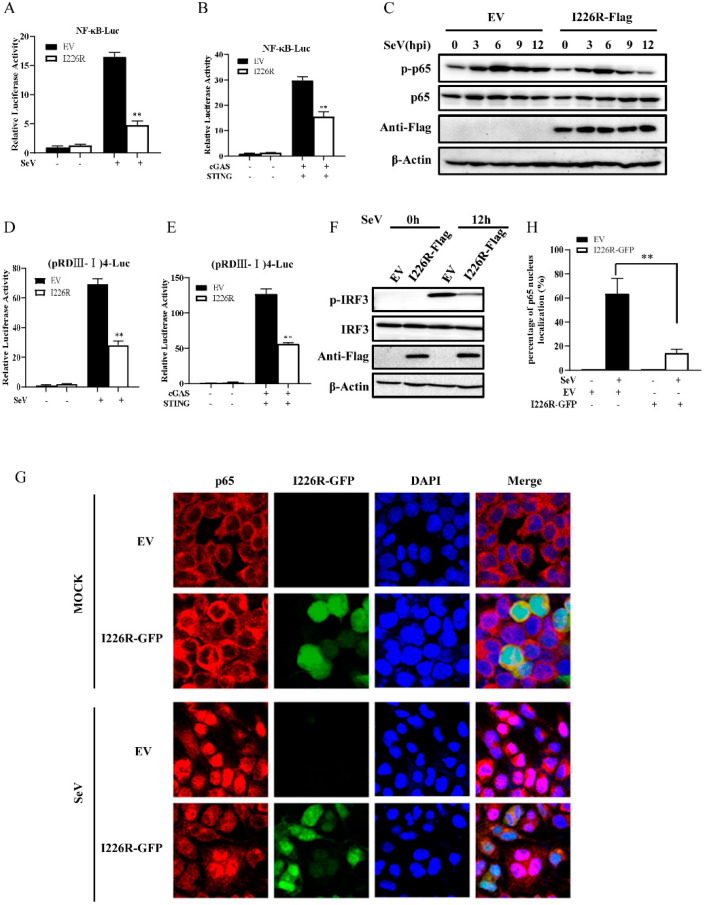
The I226R protein impairs the activation of NF-κB and IRF3 signaling: (**A**) 500 ng NF-κB-Luc, 50 ng pRL-TK, and 500 ng I226R-Flag or EV were co-transfected with 293T cells for 24 h and infected with SeV for 16 h. The NF-κB luciferase activity was measured. (**B**) 300 ng NF-κB-Luc, 30 ng pRL-TK, and 300 ng I226R-Flag or EV were co-transfected in 293T cells for 24 h with 300 ng cGAS-Flag and 50 ng STING-HA. The NF-κB luciferase activity was measured. (**C**) 293T cells were transfected 3 μg I226R-Flag or EV for 24 h and infected with SeV for indicated time. Western blotting was performed to detect the phosphorylation levels of p65. (**D**) 500 ng (pRDIII-I)4-Luc was used instead of NF-κB-Luc, as described in panel A. The IRF3 luciferase activity was measured. (**E**) 300 ng (pRDIII-I)4-Luc was used instead of 300 ng NF-κB-Luc, as described in panel B. The IRF3 luciferase activity was measured. (**F**) 293T cells were transfected with 3 μg I226R-Flag or EV and infected with SeV for 12 h. Western blotting was then performed to detect the phosphorylation levels of IRF3. (**G**,**H**) 293T cells were transfected with 3 μg I226R-GFP or EV and infected with SeV for 12 h. The cells were stained with anti-p65 Ab. Alex Fluor 594-conjugated IgG (red) was used as the secondary antibody. Cell nuclei were stained with DAPI (blue). The images were obtained by laser scanning confocal microscope using a 20× objective (**G**). Quantification of the nuclear translocation of p65 was determined by counting 100 cells from at least three independent fields (**H**). Shown are percentages of cells with p65 located in the nuclei. The results are expressed as the means ± standard deviation from three independent experiments. Statistical analysis was performed using the Student’s *t*-test. ** *p* < 0.01.

Since the I226R protein acts as an inhibitor of IFN expression, this prompted us to further investigate the effect of the I226R protein on the activation of NF-κB and IRF3. For this, we co-transfected the I226R-Flag or an EV and the NF-κB luciferase reporter. As shown in Figure 5A,B, the I226R protein strongly impaired the NF-κB luciferase activation induced by SeV and cGAS/STING. Furthermore, 293T cells were overexpressed with the I226R-Flag or an EV and infected with SeV. The phosphorylation of p65, a subunit of NF-κB, was examined. Indeed, I226R overexpression caused a significant decrease in virus-induced p65 phosphorylation, especially at 9 and 12 h postinfection (Figure 5C and Figure S1G). Moreover, we found that the I226R protein also inhibited the IRF3 luciferase activation and the phosphorylation of IRF3 in cells stimulated with SeV and cGAS/STING (Figure 5D–F).

Next, we analyzed the nuclear translocation of p65 in response to SeV infection. As shown in Figure 5G,H, the nuclear translocation of p65 was significantly impaired in I226R-overexpressing cells compared with control cells after SeV infection. Collectively, these results suggest that the I226R protein impairs the activation of NF-κB and IRF3, thereby suppressing IFN expression.

### 3.6. I226R Protein Inhibits NF-κB through Targeting the IKK Complex

**Figure 6:**
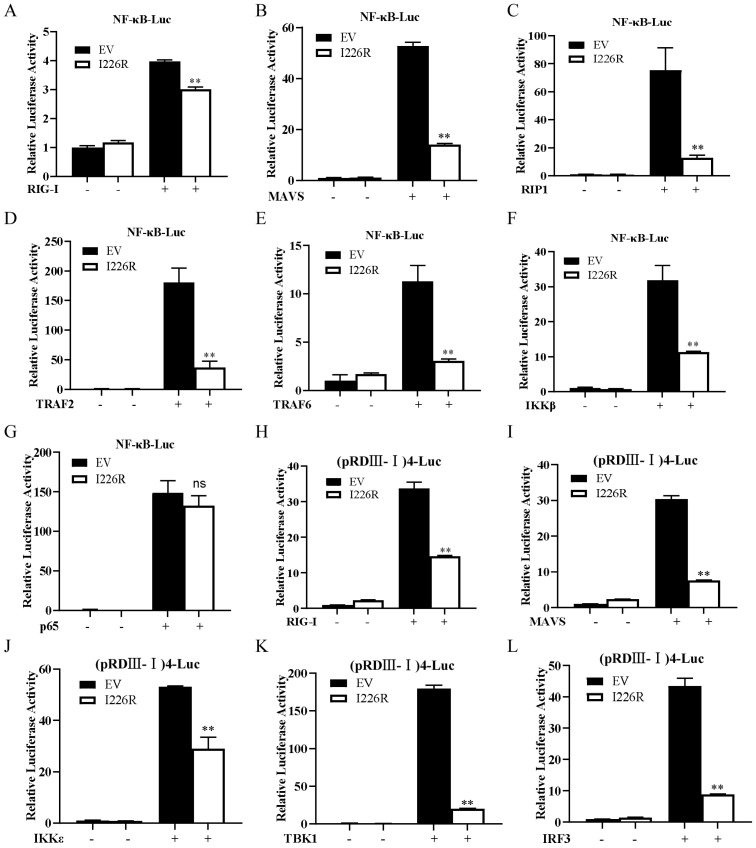
The I226R protein inhibits NF-κB through targeting the IKK complex: (**A**–**G**) 293T cells were co-transfected with 500 ng NF-κB-Luc, 50 ng pRL-TK, and 500 ng I226R-Flag or EV, along with 300 ng RIG-I (**A**), MAVS (**B**), RIP1 (**C**), TRAF2 (**D**), TRAF6 (**E**), IKKβ (**F**), or p65 (**G**). Luciferase activity was measured 24 h post-transfection. (**H**–**L**) 293T cells were co-transfected with 500 ng (pRDIII-I)4-Luc, 50 ng pRL-TK, and 500 ng I226R-Flag, along with 300 ng RIG-I (**H**), MAVS (**I**), IKKε (**J**), TBK1 (**K**) or IRF3 (**L**). At 24 h after transfection, luciferase activity was measured. All the results are expressed as the means ± standard deviation from three independent experiments. Statistical analysis was performed using the Student’s *t*-test. ** *p* < 0.01.

To uncover the mechanism by which the I226R protein regulates the activation of NF-κB, we determined the level at which I226R elicits its inhibitory effect on NF-κB signaling. The 293T cells were transfected with the NF-κB luciferase reporter and the RIG-I, MAVS, RIP1, TRAF2, TRAF6, IKKβ, or p65 expression plasmid together with either EV or I226R. The luciferase assay showed that I226R inhibited NF-κB luciferase activation mediated by RIG-I, MAVS, RIP1, TRAF2, TRAF6, and IKKβ, but not NF-κB luciferase activation induced by p65 (Figure 6A–G), implying that I226R may restrain NF-κB signaling at the level of the IKK complex.

On the other hand, we also evaluated the effect of I226R on the activation of IRF3. As shown in Figure 6H–L, the IRF3 luciferase activation induced by RIG-I, MAVS, IKKε, TBK1, or IRF3 was decreased in cells overexpressing I226R compared with control cells, suggesting that the I226R protein suppressed IRF3 activation.

### 3.7. I226R Protein Inhibits NF-κB Signaling, Likely through Regulation of NEMO

**Figure 7:**
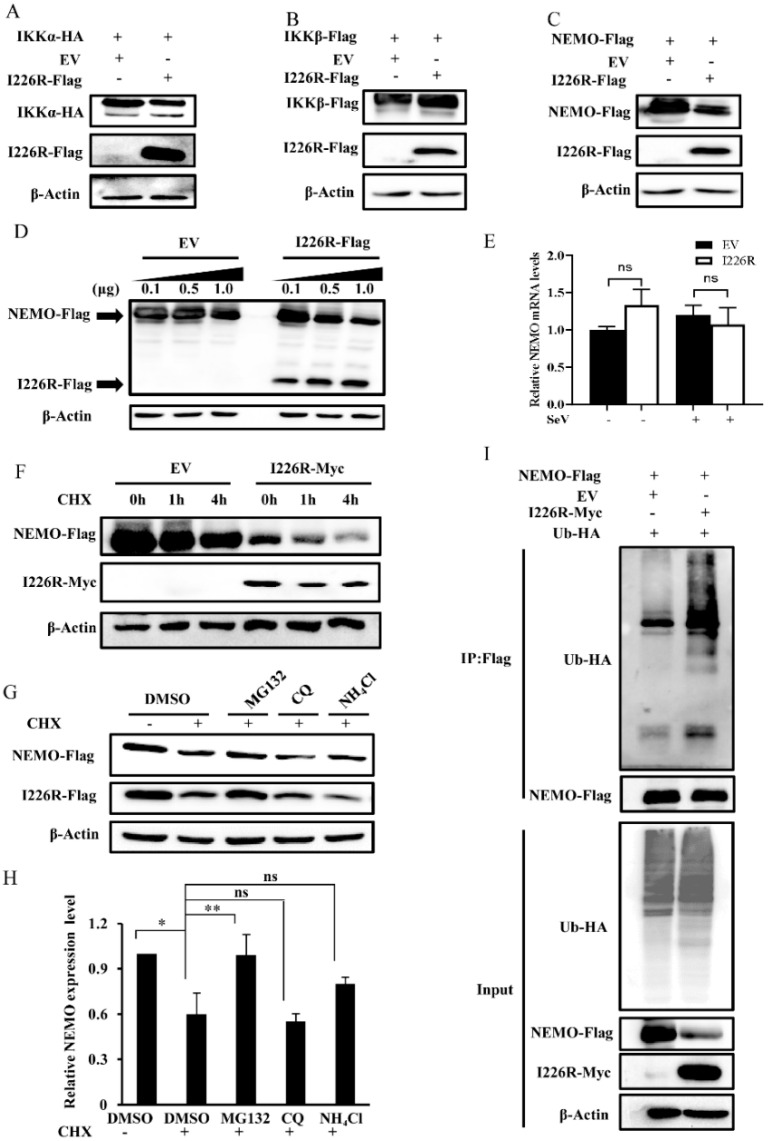
The I226R protein promotes the degradation of NEMO through enhancing its ubiquitination: (**A**–**C**) 293T cells were co-transfected with 1 μg I226R-Flag or EV and 1 μg IKKα-HA (**A**), IKKβ-Flag (**B**), or NEMO-Flag plasmids (**C**). Western blotting was then performed. (**D**) 1 μg NEMO-Flag was co-transfected with varying amounts of I226R-Flag plasmid in 293T cells. Western blotting was then performed. (**E**) 293T cells were transfected with 3 μg I226R-Flag or EV and infected with SeV for 12 h. RT-qPCR was performed to detect the mRNA levels of NEMO. (**F**) 1 μg NEMO-Flag was co-transfected with 1 μg I226R-Myc or EV and subjected to 50 μg /mL CHX treatment for indicated hours. Then, Western blotting was performed. (**G**,**H**) 293T cells were co-transfected with 1 μg I226R and 1 μg NEMO and treated with 50 μg/mL CHX and either 20 mM MG132, 50 mM CQ, 20 mM NH_4_Cl, or DMSO. Western blotting was performed to evaluate the expression of NEMO. Relative levels of NEMO were quantitated by densitometry and normalized to β-actin levels (**H**). (**I**) 293T cells were transfected with 1.5 μg I226R-Myc or EV, along with 1 μg NEMO-Flag and 1.5 μg Ub-HA. At 24 h after transfection, IP and Western blotting were performed to evaluate the ubiquitination levels of NEMO. Statistical analysis was performed using the Student’s *t*-test. * *p* < 0.05; ** *p* < 0.01.

Next, we attempted to further address the relationship between the IKK complex and the I226R protein in regulating the activation of the NF-κB pathway. The IKK complex is composed of three proteins, namely, IKKα, IKKβ, and NEMO (IKKγ). Thus, we tested the effect of the I226R protein on the expressions of IKKα, IKKβ, and NEMO. Interestingly, we found that the expression of NEMO was decreased in the presence of the I226R protein, whereas there was no significant effect of the I226R protein on the levels of IKKα, IKKβ, and CDK4 (Figure 7A–C and Figure S1H). In addition, we observed that the I226R protein could diminish the expression of NEMO in a dose-dependent manner (Figure 7D).

Since I226R affects the expression of NEMO at the protein level, we asked whether I226R could regulate the expression of NEMO at the mRNA level. As shown in Figure 7E, no significant difference in the expression of NEMO at the mRNA level was observed between I226R-overexpressing and control cells. We next examined the protein stability of NEMO after treatment with CHX, an inhibitor of protein synthesis, in I226R-overexpressing and control cells. The protein level of NEMO decreased in 1226R-overexpressing cells compared with control cells after CHX treatment (Figure 7F). These results suggest that the I226R protein promotes the degradation of NEMO protein. To further determine the pathway involved in I226R- mediated regulation of NEMO protein stability, we treated 293T cells with CHX and proteasome inhibitor MG132 or lysosomal inhibitors (CQ and NH_4_Cl). We observed that the NEMO level was restored after treatment with MG132, but not CQ and NH_4_Cl (Figure 7G,H). Finally, we evaluated whether I226R may affect the ubiquitination of NEMO. For this, 293T cells were transfected with HA-Ub and NEMO-Flag, along with I226R-Myc or an EV. The ubiquitination levels of NEMO were detected. We observed a low level of NEMO ubiquitination in cells expressing Ub and NEMO. By contrast, the ubiquitination levels of NEMO increased in cells expressing I226R, Ub-HA, and NEMO (Figure 7I), suggesting that the I226R protein may inhibit the NF-κB signaling pathway by regulating the NEMO protein, likely via ubiquitination.

## 4. Conclusions

ASF is one of the most devastating swine diseases, which can result in huge economic losses. A better understanding of the interaction between ASFV and its host paves an alternative way to treat and control such viral diseases. In recent years, scientists have been attempting to identify the ASFV proteins responsible for regulating innate immunity. Several previous researchers have suggested that ASFV could evade host innate immunity to infect host cells and replicate efficiently in the cells through several viral proteins [44]. However, limited information is available about ASFV proteins’ role in counteracting innate immunity. In addition, the precise mechanism by which these proteins regulate innate immunity is largely unknown. In this study, we screened potential ASFV proteins responsible for the regulation of innate immunity using dual-luciferase reporter assays and identified the I226R protein of ASFV as a potential suppressor of innate immunity. Our in-depth study found that I226R could inhibit the expression of IFNs by targeting NF-κB signaling. Furthermore, it was shown that the I226R protein might be involved in regulating the ubiquitination of NEMO, thereby inhibiting the NF-κB pathway. On the other hand, we observed that the I226R protein could also suppress the activation of TBK1 and IRF3, indicating that it is a multifunctional protein in its interactions with the host’s innate immune system. Together, these data reveal the function of the I226R protein and the mechanism by which it suppresses innate antiviral responses. This may provide a new theoretical basis to help in the development of new vaccines.

Innate immunity is the first line of defense against invading pathogens, and also contributes to the activation of the adaptive immune response [45]. Innate immunity activates various transcription factors to induce the expression of IFNs and pro-inflammatory cytokines to establish an antiviral response in the host. In this process, IFNs play a vital role to induce the expression of hundreds of ISGs via the JAK-STAT pathway. At present, several studies have reported that ASFV can circumvent innate immunity by targeting IFN signaling in a multitude of ways [19,20,44,46,47]. ASFV, being a DNA virus, has evolved to inhibit the cGAS/STING-dependent immune signaling, leading to the diminished production of various IFNs and ISGs [47]. ASFV encodes over 160 viral proteins, and several ASFV proteins have been characterized as inhibitors of innate immunity. For example, MGF505-7R upregulates the expression of autophagy-related protein ULK1 and promotes the degradation of STING to negatively regulate the cGAS/STING signaling pathway [19]; MGF360-12L inhibits the interaction of KPNA2, KPNA3, and KPNA4 with p65, thereby suppressing the nuclear import of NF-κB [46]. Moreover, ASFV I215L has recently been shown to inhibit type І IFN production by reducing K63-linked polyubiquitination of TBK1 [48]. Another previous study indicated that transient overexpression of I215L inhibited NF-κB and AP-1, but not IRF-3-dependent signaling [49], which is consistent with our data suggesting that overexpression of I215L had no significant effect on the activation of the IFN-β promoter by SeV in 293T cells. The different effects of I215L on innate immune signaling may be attributed to different cell lines, distinct stimulation, or varied expression levels of viral proteins in cells. In addition, DP96R protein can also downregulate the expression of type I IFNs and pro-inflammatory cytokines by targeting TBK1 [20]. Although it is known that ASFV can encode numerous proteins, the implication of most of these viral proteins in innate immunity is yet to be explored. In this study, we attempted to screen and characterize potential viral proteins encoded by ASFV in regulating the expression of IFNs. Interestingly, we found that the ASFV I226R protein inhibited the expression of IFNs induced by SeV, poly(I:C), or cGAS/STING in the 293T cell system. Moreover, overexpression of the I226R protein in 3D4/21 and PK15 also inhibited the expression of IFNs. Further study demonstrated that I226R overexpression caused a diminished expression of critical ISGs involved in antiviral responses. A previous study reported that the *I226R* gene possesses transcription initiation sites that are specifically active during a period clearly distinguishable from the early and late phases of the virus life cycle [31]. Thereafter, another report showed that the I226R protein could be expressed in the early and late stages of viral infection [32]. Notably, recent studies demonstrated that the expression of I226R was observed at 2 hpi and could be detected throughout the remaining infection cycle, although expression of I226R peaked at 8 hpi, implying that I226R is transcribed at the late stage of infection [33]. These data suggest that I226R could express during the early and late phases of viral infection, which may be associated with the host, virus strain, and challenge dose. In addition, it has been reported that I226R knockout attenuated ASFV in pigs [33]. However, the biological function of the I226R protein remains largely unknown. In the present study, we provide evidence that the I226R protein of ASFV may play a critical role in escaping from innate immune surveillance during viral infection. Notably, among the ten ASFV proteins we screened, although the expression levels of some proteins, such as I243L, were lower, they displayed some suppressive effect on the IFNβ luciferase activity, though not at the significant level. The function of these viral proteins deserves further investigation.

The NF-κB signaling pathway is a key immune signaling involved in innate immunity that induces the expression of various cytokines and IFNs. The activation of NF-κB is triggered by two types of signaling pathways: the canonical pathway and the noncanonical pathway. In this study, we mainly focused on the canonical pathway, which can be activated in many ways, including by inflammatory cytokines and PAMPs, etc., resulting in the translocation of the NF-κB transcription factor [50]. Many DNA viruses, such as alphaherpesvirus pseudorabies virus (PRV) [51], herpes simplex virus 1 (HSV-1) [52], and others, inhibit antiviral responses by targeting the NF-κB signaling pathway. Similar to other DNA viruses, some ASFV proteins have also been reported to inhibit the NF-κB signaling pathway in a different way [17,18,46]. Here, we identified a new mechanism of the ASFV I226R protein responsible for deactivating the NF-κB signaling pathway by targeting the IKK complex. We found that overexpression of the I226R protein inhibited the activation of the NF-κB reporter gene and phosphorylation of p65. To determine the potential target protein, we used dual-luciferase reporter assays. Interestingly, we observed that the I226R protein inhibited the activation of the NF-κB reporter gene induced by RIP1, TRAF2, TRAF6, and IKKβ, but not by p65 itself, suggesting that the I226R protein may target the IKK complex, a critical protein complex that functions upstream of p65. Furthermore, we found that co-transfection of the I226R protein with NEMO resulted in the decreased protein expression of NEMO, but had no effect on the expression of IKKα and IKKβ. In addition, it was shown that the I226R protein facilitated NEMO degradation after treatment with CHX, and proteasome inhibitor treatment restored NEMO accumulation. It was also shown that co-transfection of I226R and NEMO plasmids could enhance ubiquitination of NEMO. These experimental observations suggest that the I226R protein may be involved in regulating NEMO, but not IKKα and IKKβ, in the IKK complex, likely through ubiquitination. It is well known that NEMO is a key protein for the activation of the classical NF-κB signaling pathway [53]. Patients with NEMO deficiency are more susceptible to various infections with pathogens [54]. NEMO acts as an important adaptor protein in the RLR-MAVS or cGAS-STING signaling pathway. Ubiquitinated TBK1 recruits the downstream adaptor NEMO and forms the NEMO/TBK1 complex, which links the downstream nuclear factor-kappa B and interferon regulatory factors [55,56,57]. Previous reports also suggested that other viral proteins can target NEMO and impair NF-κB activation. For instance, the feline coronaviruses nsp5 protein cleaves multiple sites of NEMO [58]; porcine deltacoronavirus cleaves NEMO at conserved residue Q231 [59]; and molluscum contagiosum virus disrupts NEMO-cIAP1 interactions to suppress NF-κB activation [60]. However, the precise mechanism underlying the suppression of NF-κB by ASFV infection remains to be determined. In addition, the mechanisms accounting for I226R-mediated suppression of IRF3 activation also warrant further investigation in the future.

Ubiquitination modification plays a key role in regulating host innate immunity in response to various pathogens. For example, HSV1 could digest K63-linked ubiquitin on STING, thereby inhibiting the expression of type I IFN in the brain [61]. Newcastle Disease Virus (NDV) can promote the degradation of MAVS by enhancing ubiquitination of MAVS [62]. The nsp5 protein of PRRSV enhances STAT3 degradation through the ubiquitin-proteasome pathway [63]. Notably, IpaH9.8, a Shigella effector possessing E3 ligase activity, promotes ABIN-1-dependent polyubiquitylation of NEMO, thereby enhancing the degradation of NEMO and perturbing NF-κB activation, indicating that the ubiquitylation of NEMO could be manipulated by Shigella bacteria to modulate host inflammatory responses [64]. In this study, it was shown that ASFV I226R may be involved in regulating ubiquitination of NEMO. Although we examined the interaction between I226R and NEMO by immunoprecipitation, no direct interaction between I226R and endogenous NEMO was observed under this condition (data not shown), suggesting that other molecule(s) may be involved in the degradation of NEMO. For example, MARCH2 has been reported to directly interact with NEMO and catalyze K48-linked ubiquitination of NEMO, leading to the degradation of NEMO [65]. Therefore, it is of interest to identify whether I226R may mediate the ubiquitination of NEMO through enhancing the association of E3 ligases, such as MARCH2 with NEMO. On the other hand, we observed that the level of I226R increased in cells treated with MG132, suggesting that the expression of I226R may also be regulated in a proteasome-dependent manner in host cells. These observations demand further investigation.

In summary, our studies indicate that I226R of ASFV may act as a multifunctional protein in counteracting host innate immunity, including suppression of TBK1, IRF3, and NF-κB. I226R may be involved in regulating NEMO ubiquitination and thereby inhibits the activation of NF-κB. However, the precise strategies of ASFV to escape from innate immune surveillance require further investigation. Currently, we cannot perform experiments using live ASFV in the lab. Further studies are also required to evaluate the suppression of host innate immunity by ASFV I226R in the circumstances of wild-type or recombinant ASFV infection in vitro and in vivo in the future.

## Data Availability

All data are available in the main text or the Supplementary Materials.

## Supplementary Materials

The following supporting information can be downloaded at: https://www.mdpi.com/article/10.3390/v14030575/s1, Figure S1: (A) Relative IFN-β-Luc activity of I226R to the activity of A238L was compared by data normalization based on protein expression levels. (B,C) Overexpression of I226R protein led to diminished expression of ISG20 (B) and IFIT1 (C) induced by SeV infection. (D,E) Overexpression of I226R protein led to reduced expression of IFIT1 (D) and IFIT2 (E), in cells stimulated through transfection with poly(I:C). (F) The I226R protein caused a decrease in the expression of ISG15 upon stimulation by cGAS/STING. (G) Relative level of p-p65 as quantitated by densitometry and normalized to β-actin (represented by Figure 5C). (H) There was no significant effect of the I226R protein on the levels of CDK4.
