# Supplementary material for: I226R Protein of African Swine Fever Virus Is a Suppressor of Innate Antiviral Responses
Source: Viruses. 2022 Mar 11;14(3):575. doi: 10.3390/v14030575 (PMC8951476; doi:10.3390/v14030575)
Supplement: Supplementary file 1 [file viruses-14-00575-s001.zip › viruses-1569295-supplementary.pdf]

Supplementary figure S1

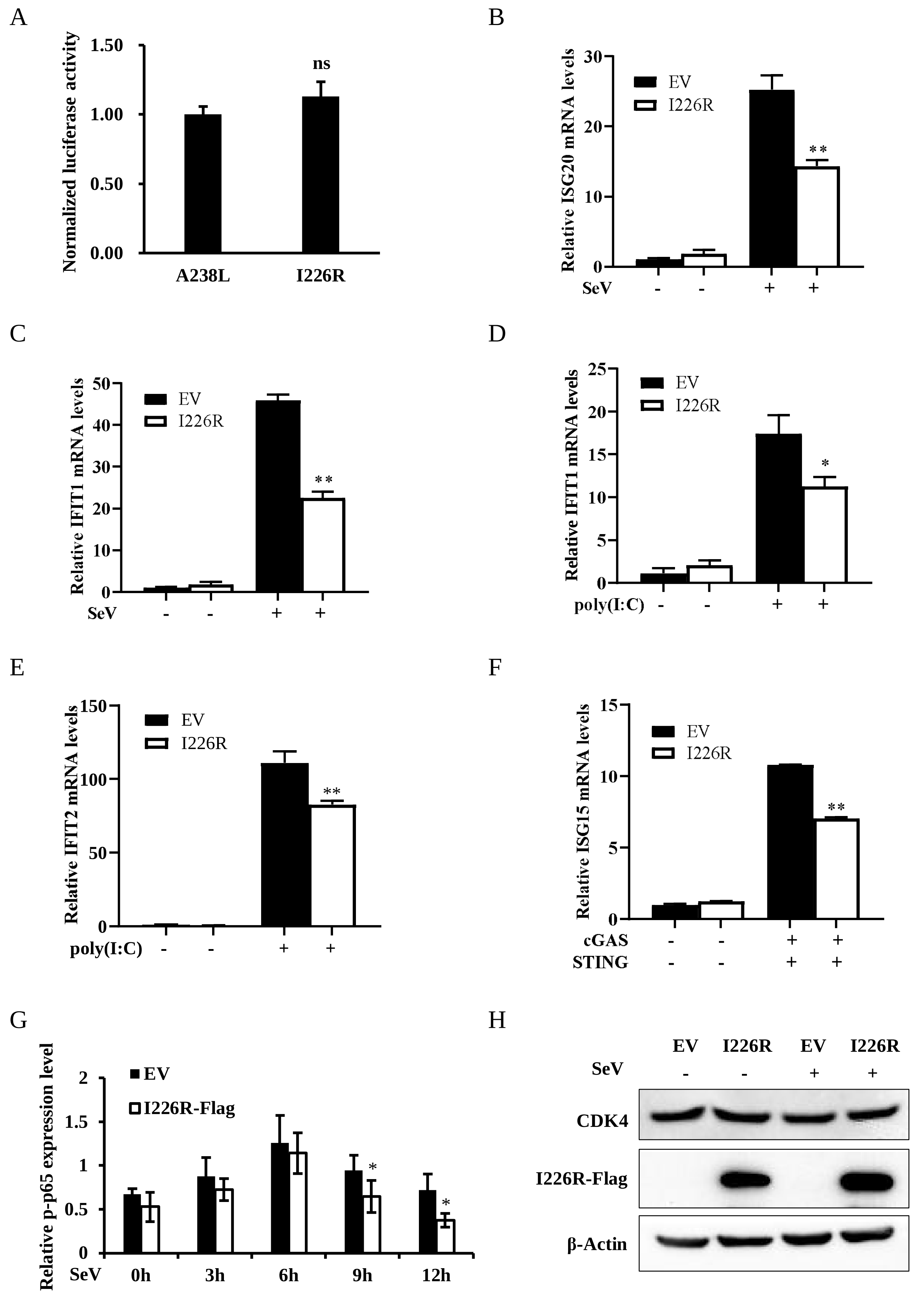

Figure S1. (A) Relative protein level of A238L and I226R was quantitated by densitometry and normalized to β–actin (represented by Figure 1A). Next, relative IFN–β–Luc activity of I226R to the activity of A238L was compared by data normalization based on protein expression levels. Shown is the average normalized luciferase activity from three independent experiments. (B,C) 293T cells were transfected with 3 μg I226R–Flag plasmid or EV for 24 h and infected with SeV for 12 h. RT–qPCR analysis were then performed to examine the mRNA levels of ISG20 (B), IFIT1 (C). (D,E) 293T cells were transfected with 3 μg I226R–Flag expression plasmid or EV for 24 h and then transfected with 1 μg/mL of poly(I:C) for 12 h. RT–qPCR analysis was performed to examine the mRNA levels of IFIT1 (D), IFIT2 (E). (F) 293T cells were co–transfected with 2 μg I226R–Flag plasmid or EV, and with 500 ng cGAS–Flag and 100 ng STING–HA plasmids for 24 h. RT–qPCR analysis was then performed to examine the mRNA levels of ISG15. (G) Relative level of p–p65 as quantitated by densitometry and normalized to β–actin (represented by Figure 5C). Shown is the average p–p65 protein level from three independent experiments. (H) 293T cells were transfected 3 μg I226R–Flag or EV for 24 h and infected with SeV for 12 h. Western blotting was then performed to detect CDK4. All the results are expressed as the means ± standard deviation from three independent experiments. Statistical analysis was performed using the Student's t-test. *, $P<0.05$; **, $P<0.01$.
